# Supplementary material for: Asynchronous magnetic resonance elastography: Shear wave speed reconstruction using noise correlation of incoherent waves
Source: Magn Reson Med. 2022 Oct 27;89(3):990–1001. doi: 10.1002/mrm.29502 (PMC9792433; doi:10.1002/mrm.29502)
Supplement: Supplementary file 1 — DATA S1 MATLAB code used to generate the numerical simulations of Figure 1 [file MRM-89-990-s002.zip › k-Wave/helpfiles/saveTiffStack.html]

saveTiffStack :: Functions (k-Wave)


# saveTiffStack

Save volume data as a tiff stack.

## Syntax

```
saveTiffStack(M)
saveTiffStack(M, filename)
saveTiffStack(M, filename, bit_depth)
saveTiffStack(M, [], bit_depth)
```

## Description

`saveTiffStack` saves 3D volume data in matrix form as a stacked tiff image. If the data is indexed as [x, y, z], each image corresponds to an [y, z] slice of the data. If no filename is given, a dialog box is invoked.

## Inputs

|  |  |
| --- | --- |
| `M` | the volume data to save |
| `filename` | filename string |
| `bit_depth` | bit depth of the tiff image (set to 8 or 16; default = 8) |

## See Also

`imwrite`
